# Serum untargeted metabolomics reveals key pathways in feline mammary carcinoma for comparative oncology

**DOI:** 10.1007/s11306-026-02430-8

**Published:** 2026-04-17

**Authors:** Hanna Carvalho de Sá, Gabriel Menezes Rodrigues, Vitor de Moraes Pina de Carvalho, Laís Pereira Silva, Massuo Jorge Kato, Alessandra Estrela-Lima, Gisele André Baptista Canuto

**Affiliations:** 1 Departmento de Química Analítica, Instituto de Química, Universidade Federal da Bahia, Salvador, 40170-110 Brasil; 2 Instituto Nacional de Ciência e Tecnologia em Bioanalítica Lauro Kubota - INCTBio-LK. Instituto de Química, Universidade Estadual de Campinas - Unicamp, Caixa Postal 6154, Campinas, São Paulo 13083-970 Brasil; 3 Research Center on Mammary Oncology (NPqOM), Federal University of Bahia, Salvador, Bahia Brazil; 4 Laboratório de Química de Produtos Naturais, Instituto de Química, Universidade de São Paulo, São Paulo, 05508-000 Brasil; 5 Department of Anatomy, Pathology and Veterinary Clinics, Federal University of Bahia, Salvador, 40170-110 Brazil

**Keywords:** Metabolomics, Breast cancer, Comparative oncology, Potential serum biomarkers, GC–MS

## Abstract

**Background and aims:**

The development of breast cancer exhibits a heterogeneous and complex character in both felines and humans, which motivates the search for serum biomarkers for diagnosis, prognosis, and therapeutic monitoring. Although the feline species is recognized as a relevant comparative model for human breast cancer, metabolomic studies in cats are still scarce. This work aimed to investigate altered serum metabolites involved in feline mammary carcinoma (FMC).

**Methods:**

Serum samples from 28 adult female cats (11 healthy and 17 with malignant mammary tumors undergoing mastectomy) were evaluated. The samples were extracted with pure methanol and derivatized (oximation followed by silylation) for GC–MS analysis.

**Results:**

Twenty-six metabolites or chemical classes were found significantly altered. The main alteration was related to the metabolism of amino acids, carbohydrates, and the tricarboxylic acid cycle, mostly with reduced serum levels in female cancer patients, suggesting high tumor uptake. Analysis of metabolic pathways revealed alterations in the metabolism of alanine, aspartate, and glutamate; arginine and proline; starch and sucrose; and butanoate metabolism.

**Conclusions:**

These findings provide preliminary evidence supporting the determination of candidate biomarkers and the mapping of disrupted metabolic pathways in FMC. Validation of the study and confirmation of the metabolite’s structural identity are critical to enable robust comparative studies and to direct the development of innovative diagnostic and therapeutic strategies.

**Supplementary Information:**

The online version contains supplementary material available at 10.1007/s11306-026-02430-8.

## Introduction

Cancer comprises a group of disorders characterized by the uncontrolled proliferation of abnormal cells and remains the second leading cause of death worldwide, accounting for approximately 9.6 million deaths annually (WHO, 2026). Among its various subtypes, breast cancer (BC) is the most frequently diagnosed malignancy in women (IARC, 2026). In veterinary medicine, mammary carcinomas are among the most common neoplasms in female cats and dogs, sharing many histological, molecular, and clinical similarities with human breast cancer. This resemblance has positioned comparative oncology as a valuable research field for elucidating cancer mechanisms and identifying translational biomarkers that benefit both human and veterinary medicine (Cassali et al., 2025).

Cancer is the leading cause of death in female cats, with feline mammary carcinoma (FMC) representing the third most common type of cancer in this species (Souza et al., 2024; Togni et al., 2013). Characterized by the development of tumors in the mammary glands of cats, FMC remains an understudied disease despite its high prevalence (Souza et al., 2024; Simeonov & Grozeva, 2024). The investigation of FMC serves as a valuable tool in comparative oncology, as domestic animals share the same environmental risk factors as humans, which are likely reflected in tumor biology (Cassali et al., 2025). FMC exhibits anatomical, biological, clinical, and histological similarities to human breast cancer, and its rapid progression allows meaningful comparison with women in terms of cancer response time and metastatic development mechanisms (Cassali et al., 2025; Zappulli et al., 2015). However, FMC diagnosis remains more limited than in humans, as the disease is often detected only at very advanced stages, resulting in an unfavorable prognosis (Zappulli et al., 2015).

Metabolomics has become an established approach in human oncology, enabling the identification of disease-specific metabolic signatures through comprehensive profiling of biofluids and tissues (Canuto et al., 2018; Santos et al., 2020; Wei et al., 2021; D’Mello et al., 2024; Silva et al., 2024). These studies have demonstrated that metabolic reprogramming in cancer involves pathways linked to glycolysis, the tricarboxylic acid (TCA) cycle, amino acid metabolism, lipid synthesis, and nucleotide turnover-features that collectively sustain tumor growth and survival. In contrast, applications of metabolomics in veterinary oncology are still emerging, with few studies focusing on feline mammary carcinoma (FMC).

Zheng et al. (2020) conducted the first serum metabolomic study in cats with mammary carcinoma using liquid chromatography-mass spectrometry (LC-MS), identifying twenty metabolites, mainly associated with glycolysis, purine metabolism, and amino acid pathways. Based on these data, Kulkarni et al. (2024) applied a machine learning model that distinguished affected from healthy cats with high accuracy. Molnár et al. (2024) later analyzed feline and canine mammary tumors using mass spectrometry-based lipidomics, revealing distinct lipid patterns between tumors and adjacent tissues, including alterations in ceramides, phosphatidylethanolamines, phosphatidic acids, and phosphatidylinositols related to tumor progression.

Despite recent advances, the metabolic profile of FMC remains largely unexplored. Thus, this study used an untargeted gas chromatography-mass spectrometry (GC-MS) approach to investigate altered serum metabolites in affected cats and to describe the main biochemical pathways involved, serving as a basis for comparative oncology.

## Methods

### Ethics statement

This work was approved by the Animal Use Ethics Committee (CEUA) of the Escola de Medicina Veterinária e Zootecnia (EMEVZ) of the Federal University of Bahia (UFBA), under protocol number 45/2022 All procedures were conducted in accordance with the Brazilian College of Animal Experimentation (COBEA). All cat owners were informed about the details of the research and project and agreed by a signed Informed Consent Form.

### Chemicals and reagents

Methanol (LC-MS grade) was purchased from JT Baker (USA). O-methoxyamine hydrochloride, N,O-bis(trimethylsilyl) trifluoroacetamide (BSTFA) plus 1% trimethylchlorosilane (TMCS), heptane (GC-MS grade), tridecanoic acid methyl ester (internal standard), and pyridine (silylation grade) were purchased from Sigma-Aldrich (Taufkirchen, Germany). Aqueous solutions were prepared using deionized water (MilliQ, Millipore, USA).

### Animals and sample collection

**Fig. 1:**
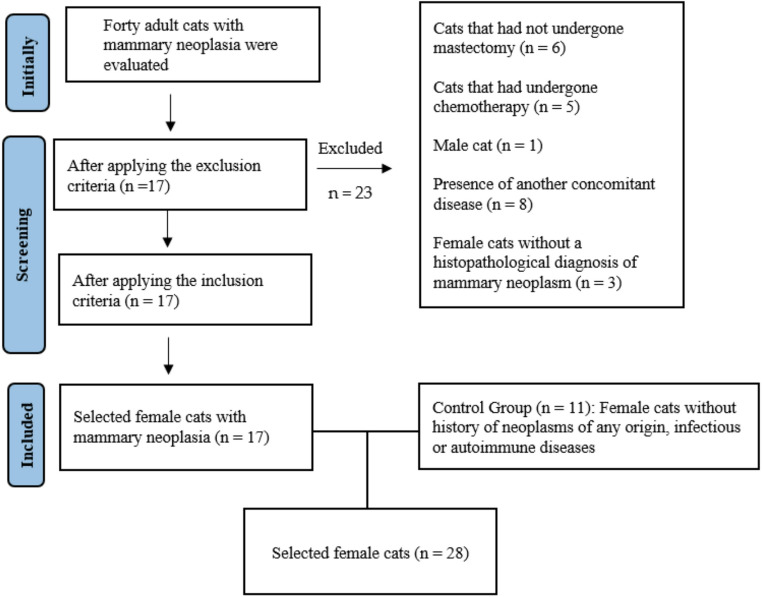
Study population flowchart: selection of felines with mammary neoplasia and controls

A total of forty female cats of different breeds with suspected mammary neoplasms, treated at the Mammary Oncology Research Center of the University Hospital of Veterinary Medicine of the Federal University of Bahia - Brazil, were initially screened for inclusion in the study. After application of the inclusion and exclusion criteria (Fig. 1), seventeen mixed-breed cats were selected to compose the experimental group (cancer group). At the same time, the remaining animals were excluded due to concurrent diseases, previous history of mammary carcinoma, or lack of histopathological confirmation. The inclusion criteria were: (a) female cats with a histopathological diagnosis of mammary neoplasm; (b) absence of another concomitant disease at the time of evaluation; (c) clinical staging from stage I onward; and (d) no previous history of mammary carcinoma surgically treated by mastectomy or lumpectomy. The exclusion criteria were: (a) cats that had not undergone mastectomy or lumpectomy and therefore did not have histopathological confirmation; (b) cats that had undergone chemotherapy within 30 days before the surgical procedure; and (c) male cats with mammary neoplasms. Information on age at neutering was available for the cancer group, with a mean age of 7.1 years. The control group consisted of clinically healthy adult female cats, initially screened across different ages; however, only mixed-breed cats met the inclusion criteria and were included in the final control group (*n* = 11). These animals were between 8 and 13 years old, with a median age of 9 years, and had no history of neoplasms of any origin, infectious or autoimmune diseases, or previous exposure to chemotherapy. The absence of concomitant diseases in both groups was assessed by means of a complete blood count, evaluation of blood smears for hematological abnormalities, including findings suggestive of hemoparasite infection, serum levels of gamma-glutamyltransferase (GGT), alanine aminotransferase (ALT), urea, creatinine, total protein and protein fractions, glucose, fructosamine, complete abdominal ultrasound, thoracic radiographs (right lateral, left lateral, and ventrodorsal views), and testing for feline immunodeficiency virus (FIV) and feline leukemia virus (FeLV).

Blood samples were collected in tubes containing a clot activator after a standardized 6-hour fasting period for both the cancer and control groups to ensure metabolic consistency between groups, and before the administration of pre-anesthetic medication, specifically to avoid interference from anesthetic agents in the serum metabolic profile of the experimental group. The samples were then centrifuged at 3,000 rpm for 10 min at 4 °C to obtain the serum. The supernatant was transferred to a microtube and immediately stored at − 80 °C until metabolite extraction.

### Clinical evaluation and mastectomy

The anamnesis was performed through a detailed evaluation of physiological parameters, clinical history, and reproductive records. Muscle mass condition was assessed according to the Muscle Mass Condition Score proposed by Michel et al. (2011). The preoperative clinical evaluation included a complete blood count, blood smear evaluation, serum urea, creatinine, gamma-glutamyltransferase (GGT), alanine aminotransferase (ALT), total protein and protein fractions (albumin and globulin), glucose, and fructosamine. Thoracic radiographic examination was performed in right lateral, left lateral, and ventrodorsal views, and a complete abdominal ultrasound was carried out to investigate distant metastases or unrelated systemic disorders. Axillary and inguinal lymph nodes were also assessed by palpation during the clinical examination. Neoplastic involvement of lymph nodes was confirmed by histopathological examination following surgical excision, particularly after unilateral mastectomy with removal of the inguinal mammary chain.

In the specific examination of the mammary glands, palpation of the entire mammary chain was performed, as well as of the inguinal and axillary lymph nodes, followed by mapping of the nodules. Clinical stage classification was performed according to tumor size (T), regional lymph node involvement (N), and presence or absence of distant metastases (M) using the TNM system. In cases with more than one nodule, each one was measured separately, and staging was performed based on the measurement of the largest tumor. All animals underwent a total unilateral mastectomy to remove the inguinal lymph node.

### Histological classification and grade

Two pathologists independently and blindly examined the original hematoxylin-eosin (HE) slides for each case, without knowledge of the prior diagnosis. When necessary, new histological sections were prepared from the original paraffin blocks and stained with HE. Tumors were classified according to the histopathological diagnosis criteria established by the World Health Organization (WHO) and further categorized based on the 2019 Consensus for the Diagnosis, Prognosis, and Treatment of Canine and Feline Mammary Tumors (Cassali et al., 2020), in parallel with the criteria proposed by the Davis-Thompson Foundation (Zappulli et al., 2015). Tumor grading followed the Nottingham histological system, as modified by Elston and Ellis (1991), which assesses tubular formation, nuclear pleomorphism, and mitotic index. Any discrepancies were resolved by discussion of multiheaded microscopy results to reach a consensus. Finally, cases with diagnoses and grades confirmed by both evaluators were included in the study.

### Follow-up and survival time

The overall survival time was defined as the period (in days) between the surgical excision of the primary tumor and the date of death due to the disease or the date of study completion. The overall survival of the animals was verified by phone calls at the end of the experiment. The survival and comparisons between groups were carried out using the log-rank (Mantel-Cox) test.

### Sample preparation

All serum samples were simultaneously thawed in an ice bath, and 100 µL of each homogenized sample was transferred to microtubes, followed by the addition of 300 µL of pure cold methanol, which was incubated at −20 °C for 30 min to precipitate the proteins. The extracts were centrifuged at 21,130 g for 10 min at 4 °C (Centrifuge 5425R, Eppendorf, Germany), and 200 µL of the supernatant was collected and transferred to vials with insert tubes. Quality control (QC) samples were prepared by mixing 100 µL of all supernatants (after protein precipitation) from the cancer and control groups, which were then divided into 200 µL aliquots and transferred to vials with insert tubes. A blank sample was prepared using the solvents and extraction procedures, replacing serum with deionized water. The extracts were dried entirely in a freeze-dryer equipment (SOLAB, Brazil) at −55 °C and − 750 mmHg. Dried samples, blank, and QCs were stored in a desiccator until derivatization.

The derivatization was performed in a two-step reaction. Firstly, 20 µL of O-methoxyamine (15 mg mL^− 1^ in pyridine) was added, followed by sonication (10 s) and vortexing (20 s). The samples were incubated overnight (17 h) in the dark at room temperature for the oximation reaction. For the silylation step, 20 µL of BSTFA with 1% TMCS was added, the mixture was homogenized and maintained at 40 °C for 30 min in a thermostatic bath (ECO SILVER RE 1225, Lauda, Brazil). The derivatized samples were resuspended in 150 µL of heptane containing the internal standard tridecanoic acid methyl ester (10 mg mL^− 1^), vortexed vigorously, and immediately analyzed in a GC-MS equipment.

### GC–MS analysis

The derivatized samples were randomly analyzed in a gas chromatography coupled to a mass spectrometry system (GCMS-QP2010 Ultra, Shimadzu). QC samples were evaluated at the beginning, every five sample sets, and at the end of the analytical sequence. The separation was performed using an HP5-MS column (30 m length, 0.25 mm i.d., 0.25 μm film of 95% dimethylpolysiloxane/5% diphenyl polysiloxane). Helium was used as carrier gas at 1.0 mL min^− 1^. The injector temperature was maintained at 250 °C, and the samples were injected in a 1:10 split using He at 5 mL min^− 1^. The initial oven temperature was 60 °C and increased to 300 °C at a rate of 10 °C min^− 1^. The MS was operated in full-scan mode (50–600 *m/z*). The electron ionization source applied − 70 eV of energy. The ion source and interface temperatures were 230 °C and 290 °C, respectively. The GC-MS Postrun Analysis (Shimadzu) software was used for instrument operation and data acquisition.

### Metabolomics data processing

The GC-MS raw data files were converted into “.abf” using Reyfics^®^ ABF converter 4.0. software (https://www.reifycs.com/abfconverter/) to be processed in MS-DIAL 5.3 (https://systemsomicslab.github.io/compms/msdial/main.html). Table S1 provides the processing steps. Metabolite annotation (levels 2 and 3) was performed based on fragmentation spectra pattern and Retention Time correlation after retention index analysis. FiehnLIB, Kasuza, and Human Metabolome Database (HMDB) spectra databases (https://systemsomicslab.github.io/compms/msdial/main.html#MSP), using a cutoff score greater than 85, were used. The most intense fragment signal in the MS spectrum established a correlation between the metabolite annotation and the associated molecular feature. The GC-MS data was normalized using the internal standard tridecanoic acid methyl ester intensity. Molecular features whose signal was less than a 5-fold change according to the Sample max/blank average ratio were removed.

The NOREVA 2.0 software (https://idrblab.cn/noreva) was used to filter the data based on the relative standard deviation (RSD) of QC samples, with a maximum limit of 30%. The software was also used for data imputation using the k-Nearest Neighbors (KNN) algorithm, correction of QCs according to the local polynomial fit regression model, log transformation, Pareto scaling, and normalization using the EigenMS algorithm prior to multivariate analysis (Karpievitch et al., 2014; Yang et al., 2020).

### Statistical and enrichment analysis

The Kaplan-Meier method was used to estimate the survival function, and the log-rank (Mantel-Cox) test was used to compare the groups. Spearman’s analysis was applied to assess correlations between parameters. The significance level adopted for all tests was *p* < 0.05, with a 95% confidence interval and two-tailed analysis. These statistical analyses were performed using SPSS^®^ 26.0 software for Windows and GraphPad Prism 8.0.2.

For metabolomics analysis, the normalized data were subjected to statistical analysis to determine the significant annotated metabolites. The MetaboAnalyst 6.0 platform (Pang et al., 2024) was used to perform statistical and enrichment analysis. Multivariate analysis was performed by building PCA (Principal Component Analysis) and OPLS-DA (Orthogonal Partial Least Squares-Discriminant Analysis) models. Significant metabolites were selected by the VIP (Variable Importance on Projection) score (VIP > 1.0) from the OPLS-DA model after checking the validation by permutation test (1,000 permutations). The univariate analysis was performed in non-normalized data, in which firstly the data normality was evaluated using the Shapiro-Wilk test using an in-house algorithm in RStudio (version 4.2). Then, according to the distribution, parametric (homogeneous- or heterogeneous-variance t-test) or nonparametric (Wilcoxon rank-sum test) tests were applied in the MetaboAnalyst 6.0 platform. Significance was determined using the False Discovery Rate (FDR), in which metabolites with FDR < 0.05 were considered significant. Significant metabolites were subjected to enrichment analysis using the KEGG database for domestic cat (*Felis catus*) metabolism, and pathways with p-value and FDR < 0.05 were considered significant.

## Results and discussion

### Clinical evaluation

The study included seventeen female cats diagnosed with mammary carcinoma, representing a population with advanced clinical presentation, which includes distant metastasis or pleural effusions, and diverse histopathological profiles. Most animals were spayed (70.59%), with a body weight ranged from 3.33 to 5.85 kg. Age distribution ranged from eight to eighteen years (median 10 years), with a higher frequency between nine and eleven years (Table S2). These values are consistent with epidemiological data reported by Zappulli et al. (2015), who described the highest incidence of feline mammary tumors in cats aged ten to twelve years.

Histological diagnosis was used to identify specific tumor cell types within the cohort. The histological grade, determined by the Nottingham system, categorized tumors into grades I-III based on tubular formation, nuclear pleomorphism, and mitotic counts. While grade III represents the highest level of cellular atypia and mitotic activity - attributes generally associated with a more aggressive biological potential - it is recognized that clinical behavior in individual cats can vary, and the disease progression may not always strictly correlate with the established grade (Elston & Ellis, 1991; Seixas et al., 2011).

Most tumors were classified as histological grade II (64.70%), suggesting an intermediate level of aggressiveness. However, the clinical stage distribution indicated a predominance of advanced disease. Stage III tumors were identified in 11 of 17 cats (64.70%), characterized by masses larger than 3 cm and/or regional lymph node involvement. Lymph node metastasis (N1) was detected in six cases (35.30%) and distant metastasis (M1) was observed in one cat (5.90%) based on imaging before treatment (Table S3).

The predominance of grade II tumors associated with stage III clinical classification reflects a population with established disease and advanced tumor presentation. This distribution mirrors the biological behavior typically reported for feline mammary carcinoma, marked by rapid progression, local invasiveness, and frequent regional metastasis. Similar findings have been described by De Campos et al. (2014), De Campos et al. (2016), and Sorenmo et al. (2013) who reported a high incidence of locally advanced and metastatic disease at the time of diagnosis. Lymph node involvement remains a consistent negative prognostic factor, associated with shorter survival and increased recurrence risk (Dagher et al., 2019; Seixas et al., 2011).

Based on the data analysis, it is concluded that the cancer group, although histologically diverse, represents a population with a well-defined clinical risk profile. The predominance of grade II tumors combined with stage III staging, i.e., tumors larger than 3 cm, characterizes a group of cats with established and considerably severe disease.

### Follow-up and survival analysis

The clinical follow-up revealed a high progression rate within the study population. At the time of study completion, 13 out of 17 cats (76.46%) had died due to causes directly related to mammary carcinoma progression, while only 4 cats (23.54%) remained alive (Table S2). The overall survival time was assessed through periodic clinical evaluations and telephone follow-ups with the guardians. This high mortality rate is consistent with the predominance of stage III tumors (64.70%) and histological grade II and III cases in our cohort, reflecting the aggressive biological behavior of feline mammary carcinoma. The overall median survival was 125 days. The Kaplan-Meier analysis showed no significant differences in survival across tumor size strata. When animals were grouped into T1, T2, and T3 categories, no significant difference was observed among the curves (log-rank, *p* = 0.7074) (Fig. S2).

### Metabolomics evaluation

GC-MS was used as the analytical platform to perform the untargeted serum metabolomic assessment of feline mammary carcinoma (FMC). Thus, twenty-eight serum samples from female cats, belonging to healthy (control, *n* = 11) and cancer (*n* = 17) groups, were investigated. During data processing, we found an outlier sample in the cancer group due to poor metabolite derivatization. Therefore, the cancer group was reduced to sixteen samples for data processing and statistical analysis. A total of 118 metabolites were initially annotated based on spectral similarity and retention time using MS-DIAL. After removing compounds with a relative standard deviation (RSD) above 30% in quality control (QC) samples, 95 annotated metabolites were retained for statistical analysis. The data matrix was normalized, and the data quality was initially evaluated by Principal Component Analysis (PCA), an unsupervised multivariate model. Figure S1 presents the PCA model (PC1 = 26.6% and PC2 = 9.6%), revealing a clear separation between the cancer and control groups and well-clustered QCs, attesting instrumental stability during data acquisition. Minor QC dispersion is attributed to individual derivatization of the QC samples, but the RSD threshold ensures the reproducibility and consistency across runs.

**Fig. 2:**
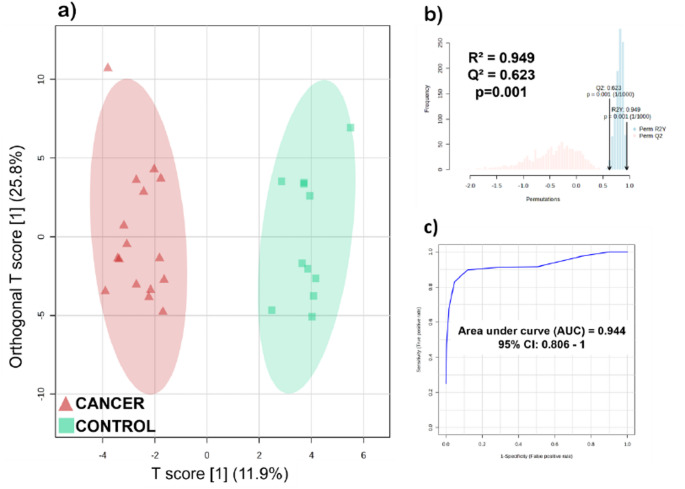
**a** Score plot for OPLS-DA model (model parameters: R^2^ = 0.949 and Q^2^ = 0.623). Cancer group is represented by red triangles and Control group is represented by green squares. **b** Permutation test plot (1,000 permutations), and **c** ROC curve of the multivariate model

To select the metabolites responsible for the differentiation between metabolism from cancer and control groups, an Orthogonal Partial Least Squares-Discriminant Analysis (OPLS-DA) model was built (Fig. 2a). The model showed strong correlation (R² > 0.949) and adequate predictive capability (Q² > 0.622). A permutation test was performed to validate the model using 1,000 permutations, and a p-value of 0.001 attests to the robustness of the model (Fig. 2b). Variable Importance on Projection (VIP) analysis indicates 26 significant metabolites or chemical classes (VIP score > 1.0) (Table 1), while receiver operating characteristic (ROC) curve analysis demonstrated high predictive capacity (AUC = 0.944; 95% CI: 0.806–1.000) (Fig. 2c).

**Table 1:** Statistically significant annotated metabolites and respective alterations in the metabolomics study of feline mammary carcinoma by GC–MS analysis

Chemical class	Metabolite/Class	VIP	FDR	FC*	AUC
Amino acids, peptides and analogues	Methionine sulfoxide	1.95	5.25 × 10^−3^	0.24	0.89
	4-Hydroxyproline	1.92	5.25 × 10^−3^	0.31	0.89
	Aspartic acid	1.87	5.25 × 10^−3^	0.61	0.89
	Glutamic acid	1.99	7.02 × 10^−3^	0.56	0.89
	Proline	1.77	1.16 × 10^−2^	0.55	0.85
	Glycine	1.43		0.48	0.73
	Creatinine	1.66		0.58	0.81
	4-Aminobutanoate (GABA)	1.51	4.22 × 10^−2^	0.82	0.82
	Leucine, norleucine, or isoleucine**	1.19		0.52	0.72
Carbohydrates and carbohydrate conjugates	Ribonic acid	2.10	2.67 × 10^−3^	0.48	0.93
	1-Kestose	1.89	7.02 × 10^−3^	0.41	0.87
	Erythronic Acid	1.81	1.16 × 10^−2^	0.60	0.87
	Isomaltose	1.05		0.69	0.76
	Gluconic acid	1.20		0.59	0.74
	Glyceric acid	1.53	4.22 × 10^−2^	0.27	0.81
	Trehalose	1.29		0.91	0.74
	Sugar alcohol (Arabitol, ribitol, or xylitol)**	1.24		0.61	0.78
	Disaccharide (Sucrose, lactose, maltose, or cellobiose)**	1.02		0.63	0.76
Carboxylic acids and derivatives	Succinic acid	1.24		0.65	0.75
	Citric acid	1.12		0.66	0.70
Glycerophosphates	Glycerol 3-phosphate	1.56	2.82 × 10^−2^	0.62	0.83
Non-metal pyrophosphates	Pyrophosphate	1.79	2.47 × 10^−2^	0.53	0.82
Short-chain hydroxy acids and derivatives	Hydroxypropionic acid	1.58		0.94	0.77
	2-Hydroxyglutaric acid	1.47	4.22 × 10^−2^	0.59	0.81
	2,4-Dihydroxybutanoic acid	1.07		0.57	0.74
Fatty acids	Palmitoleic acid	1.16		1.43	0.69

Metabolite annotation level 2, with exception of highlighted metabolites/classes (**)

*FC, fold change was calculated by the ratio of cancer to control group intensities

**Annotated class or metabolites (level 3), indistinguishable isomers with the same spectral fragmentation pattern and retention time

Univariate analyses were conducted to complement the search for discriminant metabolites. Normally distributed variables were evaluated using t-tests, whereas non-normally distributed data were analyzed with the Mann-Whitney U test. False discovery rate correction was applied, and metabolites or chemical classes with FDR values below 0.05 were considered significant. Table 1 summarizes the metabolites and classes with statistically significant differences. Fold change (FC) was calculated as the ratio of the mean intensities between cancer and control samples, in which FC > 1.0 indicates an increase in metabolite levels in the cancer group, and FC < 1.0 indicates a decrease.

Most metabolites exhibited decreased concentrations in the cancer group, suggesting consumption or metabolic redirection within tumor-associated pathways. Only palmitoleic acid was increased in the cancer group (FC = 1.43), consistent with enhanced lipogenesis previously linked to neoplastic processes. To evaluate their diagnostic relevance, the area under the curve (AUC) was calculated for each significant metabolite using receiver operating characteristic (ROC) analysis (Table 1). Ribonic acid was classified as an excellent predictor (AUC = 0.93), followed by a subset of thirteen metabolites with good predictive performance (0.8 > AUC < 0.9), and thirteen metabolites classified as moderate predictors (AUC below 0.8) (Table 1). These results suggest that specific amino acid and carbohydrate derivatives may serve as exploratory biomarkers to distinguish cats with mammary carcinoma from healthy individuals, in agreement with previous observations in other metabolomic studies of cancer metabolism (Xia et al., 2013).

As shown in Table 1, more than 60% of the significant annotations belong to the classes of amino acids, peptides and analogues (32%) and carbohydrates and carbohydrate conjugates (32%). Also, it is important to highlight that metabolite annotation in GC-MS is challenging because isomers produce identical fragmentation spectra and have practically indistinguishable retention times (Mastrangelo et al., 2015). Consequently, some isomer metabolites, such as leucine, norleucine, and isoleucine, and certain carbohydrates and carbohydrate conjugates (such as sugar alcohol and disaccharide), may not be distinctly annotated. These compounds are then grouped as a single molecular feature, with annotation level 3 (Table 1).

**Fig. 3:**
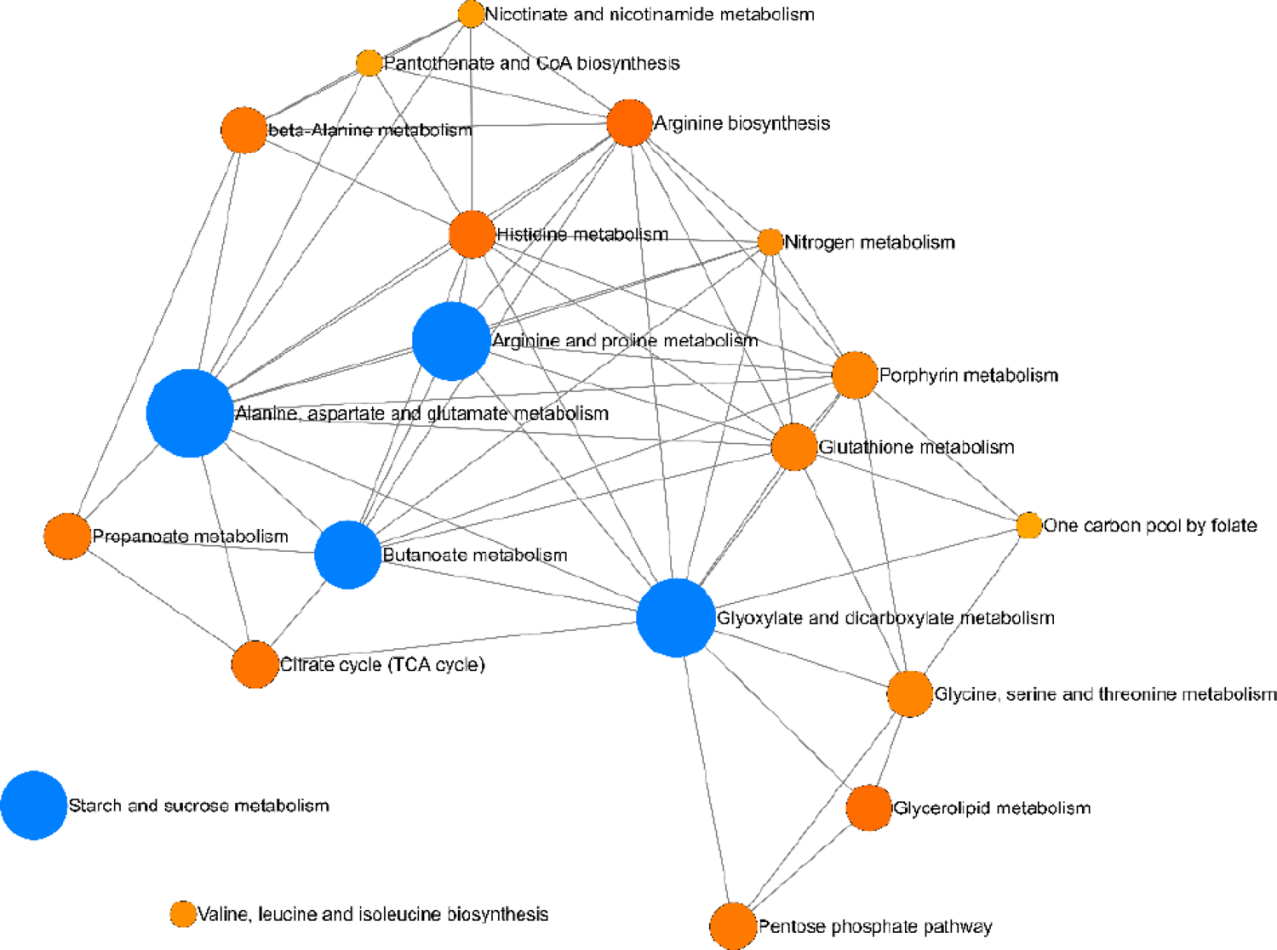
Network view from enrichment analysis. Highlighted pathways are significant altered Alanine, aspartate and glutamate metabolism (FDR = 5.85 × 10^− 4^), glyoxylate and dicarboxylate metabolism (FDR = 9.68 × 10^− 3^), butanoate metabolism (FDR = 9.68 × 10^− 3^), arginine and proline metabolism (FDR = 9.68 × 10^− 3^), and Starch and Sucrose metabolism (FDR = 1.32 × 10^− 2^)

A metabolic enrichment analysis was performed to better understand the alterations in cancer metabolism. Thus, annotated metabolites were evaluated by pathway analysis in MetaboAnalyst 6.0 using the KEGG (Kyoto Encyclopedia of Genes and Genomes) database. The enrichment analysis revealed significant alterations in alanine, aspartate and glutamate metabolism (FDR = 5.85 × 10^− 4^), glyoxylate and dicarboxylate metabolism (FDR = 9.68 × 10^− 3^), butanoate metabolism (FDR = 9.68 × 10^− 3^), arginine and proline metabolism (FDR = 9.68 × 10^− 3^), and starch and sucrose metabolism (FDR = 1.32 × 10^− 2^) (Table S4). Figure 3 presents a network visualization of these results, in which the significantly altered pathways (blue dots) are correlated with other pathways affected by alterations in the significant metabolites.

The interconnected metabolic pathways (Fig. 3) indicate high amino acid turnover and active nitrogen handling in tumor-bearing cats, consistent with feline serum findings that implicate alanine-aspartate-glutamate and related energy pathways (Zheng et al., 2020) and with FMC classification models that prioritize glutamine/glutamate and arginine pathways (Kulkarni et al., 2024). The pattern aligns with canine mammary tumor literature describing glutamine-proline flux, redox support, and lipid/energy reprogramming as recurring features of tumor biology (Tamarindo et al., 2023), and with human breast cancer syntheses that place glutamine/glutamate and pentose phosphate activity at the core of metabolic subtypes and anabolic/redox demands (Perestrelo & Luís, 2025). Within this framework, altered glutamate metabolism and ammonia recycling in our cohort indicate an adaptive program that supports anaplerotic metabolism and maintains redox balance, a conserved mammary carcinoma phenotype across species.

### Clinical staging evaluation

**Fig. 4:**
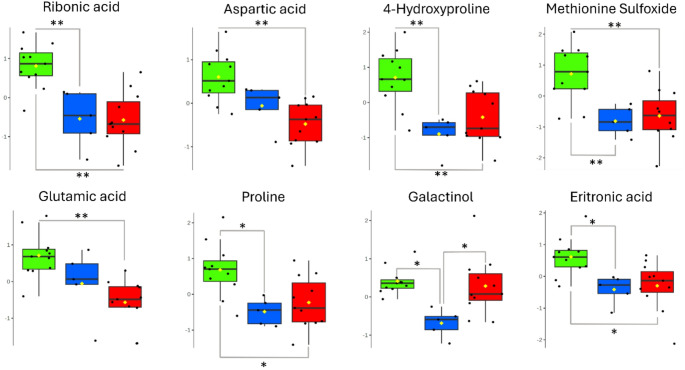
Box plots of significantly altered metabolites by ANOVA analysis comparing different clinical stages in feline mammary carcinoma evaluated by untargeted metabolomics, in which (*) indicates p-value < 0.05 and (**) indicates p-value < 0.01. Green box - Control; Blue box - Stages I-II; Red box - Stages III-IV. Yellow dots - Mean of the group; Black dots - samples

The cats in this study displayed variability in histological subtype, tumor grade, and clinical stage (Tables S2 and S3), which likely contributed to metabolic diversity. An analysis of variance (ANOVA) comparing early (I–II) and advanced (III–IV) stages revealed eight metabolites with significant differences (Table S5; Fig. 4). Ribonic acid showed the highest F-value (14.29; FDR = 0.01), with consistent reductions in both tumor groups relative to controls, indicating an early disturbance in carbohydrate oxidation and pentose phosphate activity. Comparable decreases were observed for 4-hydroxyproline, methionine sulfoxide, proline, and erythronic acid, suggesting enhanced amino acid utilization to sustain biosynthetic and antioxidant processes within tumor tissue. These findings align with serum metabolomic studies in cats that report disruptions in alanine, aspartate, and glutamate pathways linked to energy imbalance and redox control (Zheng et al., 2020; Kulkarni et al., 2024).

Aspartic and glutamic acids showed more pronounced reductions in advanced stages (III–IV), suggesting their involvement in tumor progression. Both metabolites participate in anaplerotic replenishment of the tricarboxylic acid cycle and glutathione synthesis, processes that support energy production and oxidative stability in proliferating cells. Similar mechanisms have been described in canine mammary tumors, where glutamine-proline cycling and nitrogen redistribution sustain anabolic and antioxidant demands (Tamarindo et al., 2023). Parallel alterations in human breast cancer further indicate that glutamine and pentose phosphate metabolism define metabolic subtypes that correlate with tumor aggressiveness and treatment resistance (Perestrelo & Luís, 2025).

In this cohort, most tumors were classified as histological grade II (64.70%). Only 6 of 17 cats had metastasis in regional lymph nodes, and 1 of 17 had distant metastasis on preoperative imaging (Table S2).

### Biological interpretation

Our results indicate a significant decrease in the levels of nine amino acids and analogues (Table 1), as well as changes in three metabolic pathways related to amino acid metabolism (Fig. 3). Amino acids have been extensively studied in cancer metabolism, especially in humans, with controversial results (Proenza et al., 2003; Lai et al., 2005; Eniu et al., 2019). Some studies report that cancer patients often have reduced amino acid availability due to cancer-related mal-nutrition and increased nitrogen demand by cancer cells (Lai et al., 2005). To date, no work has been found investigating the concentration of amino acids in cats. However, some studies evaluated dog plasma and human serum and reported a decrease in the concentration of this class of metabolites (Eniu et al., 2019; Azuma et al., 2012). These findings indicate that some metabolic changes, specifically in amino acid levels, may exhibit similar patterns across species.

Among the altered metabolic pathways (Fig. 3), alanine, aspartate, and glutamate metabolism were significantly changed, as indicated by decreased levels of aspartate, glutamate, GABA, glycine, succinate, and pyrophosphate (Table 1). A dysregulation in these metabolic pathways may be correlated with the cell proliferation of the cancer environment, in which metabolites from these pathways are necessary for the development of tumors, through protein synthesis and as precursors of different biosynthetic pathways such as purines and nucleotides (Lai et al., 2005; Vettore et al., 2020).

Arginine and proline metabolism was also significantly altered (FDR = 9.68 × 10^− 3^); this change occurred mainly due to the decrease of proline (FC = 0.18) in the cancer group. Proline is an important metabolite that contributes to different cellular pathways, such as protection against stress, apoptosis and metabolism of cancer cells, and in addition to it, 4-hydroxyproline was also decreased in the cancer group (FC = 0.13). The 4-hydroxyproline is the product of proline hydroxylation after interaction with collagen, a potential carcinogenic biomarker that contributes to cancer fibrosis (Amiri-Dashatan et al., 2022). This same type of metabolic alteration was previously observed in human plasma, in which the authors correlated that greater catabolism of this compound would be related to the promotion of tumor growth and the development of metastases (Wei et al., 2021).

**Fig. 5:**
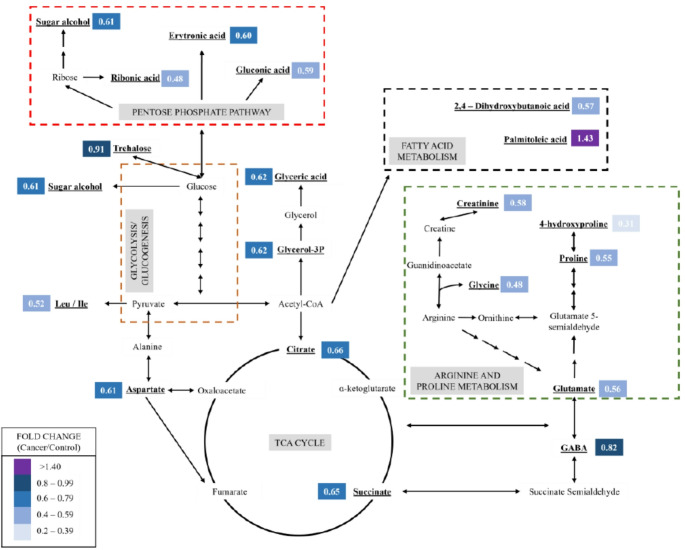
Representation of the metabolic pathways related to the significantly altered metabolites. Leu - Leucine; Ile - Isoleucine; GABA - gamma-aminobutyric acid

Figure 5 shows a representation of the metabolic changes in the highlighted amino acid pathways, along with some pathways correlated with other altered metabolites. The hypothesis raised here is that the decrease in amino acids levels in the serum is related to greater absorption of these species by tumor cells. An et al. (2022) previously correlated the decrease in glutamate and cysteine in plasma samples of women with breast cancer with the increase in the level of these metabolites in the tissue. A similar mechanism is suggested by recent feline-specific tissue proteomic findings in Paphussaro et al. (2024), who reported marked upregulation of phenylalanine-tRNA ligase (FARS2) in metastatic and high-grade FMC tissues. Since aminoacyl-tRNA ligases are essential for protein biosynthesis, their overexpression indicates increased translational activity within the tumor microenvironment, reinforcing the hypothesis that enhanced amino acid utilization by tumor cells contributes to the depletion of circulating amino acid precursors observed in our GC-MS profile. In contrast, Zheng et al. (2020) reported, using HILIC-MS, increased levels of metabolites associated with alanine, aspartate, and glutamate metabolism. These discrepancies highlight the challenges of cross-study comparisons, driven by differences in analytical platforms, biological matrices, disease stage, cohort composition, and sample size (Eniu et al., 2019).

Regarding sample heterogeneity, specifically in the Zheng et al. (2020) study, a more limited controlled sample set (*n* = 6) consisting of a single breed (Chinese pastoral cat) of unspayed cats was used. Consequently, their findings may reflect metabolic alterations specific to that group. In contrast, our study, despite using strict inclusion criteria, includes a more diverse population, aiming to provide a broader perspective on the metabolic changes caused by the tumor. Also, some species-specific or study-dependent variations were observed. While our study and some human reports indicate a systemic depletion of serum amino acids, other feline investigations using different analytical platforms (e.g., LC-MS) have reported increases in similar metabolites. These differences may be attributed to the typically advanced clinical stage of feline patients at diagnosis (stage III/IV), which may intensify the ‘sink effect’ of the tumor on host nutrient pools more severely than is often captured in human early-detection cohorts.

Additionally, the different analytical techniques employed allow access to different portions of the metabolome, as well as variations in common metabolites. In GC-MS, the derivatization step, along with sample preparation, can affect metabolite levels. Conversely, LC-MS analyses without proper spectral confirmation can lead to erroneous or ambiguous findings and, consequently, divergent biological interpretations. By organizing these findings, we demonstrate that while the fundamental metabolic ‘reprogramming’ is conserved across species, the magnitude and direction of serum shifts are influenced by species-specific disease progression and analytical sensitivity.

GABA, an inhibitory neurotransmitter in the central nervous system, has been a metabolite of interest in cancer studies. Some studies indicate that high levels of this neurotransmitter in tumor cells are associated with decreased tumor proliferation and breast cancer metastasis (Brzozowska et al., 2017; Jayachandran et al., 2023). A follow-up study of the survival rate of women with breast cancer correlated high levels of GABA in patients with better prognosis and increased overall survival (Brzozowska et al., 2017). In contrast, decreased levels of GABA could be associated with an increased risk of death. In our study, GABA was reduced in the cancer group (FC = 0.29; Table 1), demonstrating consistency with another study that investigated targeted biomarkers in male breast cancer patients (Ahmed et al., 2021).

Citrate and succinate metabolites of the tricarboxylic acid cycle (TCA) significantly decreased (FC = 0.41 and FC = 0.21, respectively), hypothesizing changes in energy metabolism. The presence of cancer cells directly affects energy metabolism because of the Warburg effect, in which glycolysis contributes more to ATP generation than mitochondrial respiration (Eniafe & Jiang, 2021). A decrease in citrate levels has already been observed in thyroid cancer and correlated with a greater consumption of this metabolite by cancer cells (Du et al., 2021), suggesting that the depletion of TCA cycle intermediates occurs due to their capture by tumor. Furthermore, our results indicate a decrease in carbohydrates in the cancer group, with nine metabolites significantly altered (Table 1), which may be related to the tumor’s higher energy demand. The altered carbohydrates and their derivatives belong to the subclasses of sugar acids (ribonic acid, erythronic acid, gluconic acid), monosaccharides, disaccharides, and sugar alcohols.

Sugar acids are produced by the oxidation of aldoses and hexoses, and these metabolites are derived from the pentose phosphate pathway (PPP). All these metabolites showed reduced levels in the cancer group (ribonic acid FC = 0.12; erythronic acid FC = 0.14; gluconic acid FC = 0.43). A decrease in these metabolites may indicate dysregulation of the PPP, a metabolic pathway that produces NADPH, a reducing species that neutralizes oxidative stress damage and supports lipogenesis. Among these compounds, erythronic acid shows a strong correlation with the PPP and has been proposed as a potential metabolite for investigating alterations in this cancer-related metabolic pathway (Zhang et al., 2023). In this context, a reduction in serum levels of this metabolite could be associated with increased PPP activity, providing more nutrients to the tumor cells.

Additionally, the reduction of erythronic acid in serum has been associated with cachexia in cancer patients, a syndrome characterized by body weight loss (More et al., 2024). In feline oncology, Baez et al. (2007) reported that 91% of cats with cancer showed evidence of muscle loss, which has been considered a negative prognostic factor for survival. In the present study, muscle mass condition (MMCS) varied among cats in the cancer group, with MMCS 1/3 observed in 2 animals, MMCS 2/3 in 8 animals, and MMCS 3/3 in 7 animals. These findings suggest that part of the cohort exhibited reduced muscle mass, which may be consistent with a catabolic state associated with neoplastic disease. However, further investigation is required to determine whether these alterations are indicative of cachexia in felines and how they relate to the observed metabolic profile.

Although cancer metabolism is often discussed primarily in the context of glycolysis, other pathways, including amino acid utilization and host protein mobilization, also play central roles in tumor progression and cancer-associated catabolism (Porporato, 2016). In this context, the depletion of circulating amino acids and carbohydrates observed in the present study should be interpreted cautiously, since these alterations may reflect not only tumor-driven metabolic reprogramming, but also systemic effects related to nutritional status and muscle loss. Cancer cachexia is characterized by progressive wasting of skeletal muscle, with or without loss of adipose tissue, and inflammatory mediators such as TNF-α, IL-6, and IL-1 are known to contribute to muscle proteolysis and metabolic imbalance (Setiawan et al., 2023; Agca & Kir, 2024). This issue is particularly relevant in feline oncology, as muscle loss has been reported in most cats with cancer, and poorer body condition has been associated with shorter survival (Baez et al., 2007). Therefore, the metabolic profile observed here may reflect a combination of tumor-associated metabolic demand and host systemic catabolism, which should be considered when interpreting reductions in amino acids and energy-related metabolites.

Kashi and Parastar (2024) observed a decrease in trehalose and mannose levels and identified these metabolites as possible biomarkers for breast cancer. Trehalose is a storage saccharide of glucose units that acts in protecting against oxidative stress and has been suggested as a potential therapy to minimize the effects of chemotherapy treatments (Chaitanya et al., 2021). Table 1 also showed a decrease in the level of saccharides.

The metabolic perturbations identified in this study highlight both conserved and distinct compared with those in human breast cancer. Among the conserved signatures, dysregulation of alanine, aspartate, and glutamate metabolism has also been reported in human breast cancer metabolomic studies, and reduced circulating aspartate has been described as a characteristic metabolic feature in affected women (Yang et al., 2020; Xie et al., 2015). Alterations related to central carbon metabolism and the tricarboxylic acid cycle have likewise been reported in human breast cancer, supporting the concept of systemic metabolic reprogramming to sustain tumor growth (Subramani et al., 2022; Silva et al., 2019).

In addition, the reduction in GABA observed in our feline cohort is noteworthy, given human data showing that lower GABA levels are associated with poorer overall survival in breast cancer patients, supporting its potential relevance as a comparative metabolic marker (Brzozowska et al., 2017). Nevertheless, some metabolic features may also reflect species-specific biology, differences in sample type, tumor subtype distribution, disease stage, and analytical platform, and therefore should be interpreted cautiously in a comparative oncology framework.

Alterations in alanine, aspartate, and glutamate metabolism have also been reported in human breast cancer metabolomic studies, supporting the biological relevance of this pathway and suggesting potential metabolic vulnerabilities that may be explored therapeutically (Yang et al., 2020; Xie et al., 2015; Subramani et al., 2022). These alterations are similar to findings in human breast cancer studies, reinforcing the potential of the feline model for translational research. Understanding metabolism and evaluating new treatments in animal models that are more faithful to human reality are ethically advantageous and reduce the probability of failure in the search for new therapeutic targets (Gherman et al., 2024). However, some factors should be considered, such as pronounced depletion, which likely reflects the unique physiological profile of cats, who are more significantly impacted by amino acid and carbohydrate imbalances due to their obligate carnivorous nature. Consequently, felines may be more acutely affected by tumor nutrient sequestration than omnivorous species such as humans, thereby amplifying the systemic metabolic signature of the disease (Ahn & Yun, 2026; Li & Wu, 2023).

In summary, the metabolic analysis reveals a profound reprogramming of central metabolic pathways in feline mammary carcinoma, highlighted by a marked depletion of metabolites associated with central metabolism. However, the elucidation of the specific functional role of each significantly altered metabolite in the context of mammary carcinoma remains poorly understood, underscoring the need for future investigations to evaluate the function of these compounds and their involvement in the clinical implications of cancer. Larger sampling and evaluation of metabolic changes using analytical techniques complementary to GC-MS, in addition to confirmation of structural identity with internal analytical standards, may assist in validating and expanding metabolic coverage. Here, we obtained initial information that indicated FMC behavior resembles that of human breast cancer, suggesting that studies in female cats can advance oncology research.

In this context, after validation and confirmation at level 1, the annotated metabolic signatures offer promising avenues for clinical application. For diagnostic purposes, ribonic acid stands out as a promising candidate for early screening, given its high predictive performance (AUC = 0.93) and reduction even in early clinical stages. Other metabolites, such as erythronic acid and 4-hydroxyproline, also demonstrated good diagnostic accuracy and could be integrated into a multi-metabolite screening panel. Regarding prognostic applications, the more pronounced depletion of aspartic and glutamic acids in advanced stages (III-IV) suggests that these compounds may reflect metabolic tumor burden and disease progression. Furthermore, the development of a multivariate diagnostic panel of biomarkers could enhance early detection of FMC through less invasive samples, providing a valuable tool for veterinary screening. Ultimately, translating these analytical findings into clinical practice could significantly improve the management and survival time of feline patients.

## Conclusion

This exploratory, proof-of-concept study demonstrates that feline mammary carcinoma is associated with profound systemic metabolic reprogramming, characterized by the depletion of key amino acids and carbohydrates. Using untargeted GC-MS metabolomics, we observed several alterations in the blood serum metabolome of affected cats, particularly decreased levels of metabolites and chemical classes linked to central metabolic pathways, including those involved in energy production and oxidative stress. Notably, dysregulation of pathways such as alanine, aspartate, and glutamate metabolism reveals conserved metabolic signatures, supporting the translational relevance of the feline model in comparative oncology.

While the limited cohort size and histological heterogeneity warrant cautious interpretation, these findings offer preliminary insights and establish a metabolic framework for future investigations. Importantly, the results are consistent with metabolic alterations previously observed in human breast cancer, reinforcing the value of this approach for translational research. Collectively, these discoveries facilitate a deeper understanding of mammary oncology and may ultimately contribute to the development of innovative diagnostic and therapeutic strategies benefiting both human and veterinary medicine.

## Electronic Supplementary Material

Below is the link to the electronic supplementary material.

Supplementary Material 1

## Data Availability

Raw data are publicly available in the link: https://github.com/gicanuto/GC-MS-Feline-Mammary-Carcinoma-.
