# Supplementary material for: Serum untargeted metabolomics reveals key pathways in feline mammary carcinoma for comparative oncology
Source: Metabolomics. 2026 Apr 17;22(3):53. doi: 10.1007/s11306-026-02430-8 (PMC13090259; doi:10.1007/s11306-026-02430-8)
Supplement: Supplementary file 1 — Supplementary Material 1 [file 11306_2026_2430_MOESM1_ESM.pdf]

Supplementary Information

Serum Untargeted Metabolomics Reveals Key Pathways in Feline Mammary Carcinoma for Comparative Oncology

Hanna Carvalho de Sá[a,b] [0000-0003-4064-6191], Gabriel Menezes Rodrigues[c] [0000-0001-9737-812X], Vitor de Moraes Pina de Carvalho[c] [0000-0003-3315-3807], Laís Pereira Silva[c] [0000-0002-3564-6077], Massuo Jorge Kato[d] [0000-0002-3315-2129], Alessandra Estrela-Lima[c,e] [0000-0002-9879-7999], Gisele André Baptista Canuto[a,b*] [0000-0002-6616-3705]

[a]Departmento de Química Analítica, Instituto de Química, Universidade Federal da Bahia, Salvador 40170-110, Brasil.

[b]Instituto Nacional de Ciência e Tecnologia em Bioanalítica Lauro Kubota - INCTBio-LK. Instituto de Química, Universidade Estadual de Campinas - Unicamp, Caixa Postal 6154, Campinas, São Paulo, 13083-970, Brasil.

[c]Research Center on Mammary Oncology (NPqOM), Federal University of Bahia, Salvador, Bahia, Brazil.

[d]Laboratório de Química de Produtos Naturais, Instituto de Química, Universidade de São Paulo, São Paulo 05508-000, Brasil.

[e]Department of Anatomy, Pathology and Veterinary Clinics, Federal University of Bahia, Salvador 40170-110, Brazil.

*Corresponding author: gih.canuto@gmail.com or gisele.canuto@ufba.br

Table S1. MS-DIAL parameters for GC-MS data processing.

MS-DIAL parameters		
**Measurement parameters**	Ionization type	Hard ionization (GC-MS)
	Separation type	Chromatography
	Data type	Centroid data
**Data collection**	Mass range	40-600
	Retention time range	5-30
	Number of threads	3
**Peak detection**	Minimum peak height	10000
	Mass slice width	0.5
	Mass accuracy for centroiding	0.5
	Smoothing method	Linear weight moving average
	Smoothing level	2
	Average peak width	10
**Spectrum deconvolution**	Sigma window value	0.5
	EI spectra cut off	10
**Identification/ Annotation**	Retention time setting	RT
	RT tolerance	0.5
	m/z range begin	40
	m/z range end	600
	m/z tolerance	0.5
	EI similarity cut off	70
	Dot product score cut off	60
	Identification score cut off	70
**Alignment**	Reference file	QC5
	RI or RT	RT
	Retention time tolerance	0.1
	EI similarity tolerance	70%

EI, Electron Ionization; RT, retention time; RI, retention index.

Table S2. Clinical and pathological data on feline mammary carcinoma.

SAMPLE NAME	DIAGNOSIS	T	N	M	Clinical Stage	Age (Years)	Survival (days)	Neutered
DS03	Papillary adenocarcinoma	T3	N0	M0	III	9	222	Yes
DS04	Cribiform Carcinoma	T3	N1	M0	III	10	96	No
DS05	Cribiform Carcinoma	T3	N0	M0	III	11	318	No
DS07	Cribiform Carcinoma	T2	N0	M0	II	15	210	Yes
DS15	Cribiform Carcinoma	T2	N0	M0	II	12	735	Yes
DS16	Cribiform Carcinoma	T3	N1	M0	III	9	120	No
DS19	Cribiform Carcinoma	T3	N1	M0	III	9	117	No
DS17	Cribiform Carcinoma with solid areas	T3	N0	M0	III	9	184	Yes
DS06	Papillary Carcinoma	T3	N0	M0	III	8	145	Yes
DS11	Papillary Carcinoma	T3	N1	M0	III	11	79	Yes
DS13	Papillary Carcinoma	T2	N0	M0	II	8	163	No
DS20	Papillary carcinoma with invasive micropapillary areas	T3	N1	M1	IV	10	82	Yes
DS08	Microinvasive papillary carcinoma	T1	N0	M0	I	11	171	Yes
DS18	Solid papillary carcinoma with cribriform area	T3	N0	M0	III	15	1002	Yes
DS01	Tubulopapillary carcinoma	T3	N0	M0	III	18	100	Yes
DS09	Tubulopapillary carcinoma	T2	N1	M0	III	9	2	Yes
DS12	Tubulopapillary carcinoma	T1	N0	M0	I	11	120	No

T: Tumor size; N: Regional lymph node involvement; M: Distant metastasis; T1: T < 2 cm; T2: 2 cm > T > 3 cm;

T3: T > 3 cm; N0: Absence of nodal metastasis; N1: Presence of nodal metastasis; M0: Absence of distant metastasis

M1: Presence of distant metastasis

Table S3. Clinical and pathological characteristics of female cats with malignant mammary tumors

Clinical-Pathological Characteristics	Parameters	Experimental group (n=17)
Age (years)	Median	10
	Range (Min - Max)	8 - 18
Weight (kg)	Range (Min - Max)	3.33 - 5.85
Tumor size	< 2 cm	2 (11.76%)
	2 – 3 cm	4 (23.54%)
	> 3 cm	11 (64.70%)
Nodal Metastasis	N0 (Absent)	11 (64.70%)
	N1 (Present)	6 (35.30%)
Distant Metastasis	M0 (Absent)	16 (94.10%)
	M1 (Present)	1 (5.90%)
Clinical stage	Stage I	2 (11.76%)
	Stage II	3 (17.64%)
	Stage III	11 (64.70%)
	Stage IV	1 (5.90%)
Histological grade	Grade I	2 (11.76%)
	Grade II	11 (64.70%)
	Grade III	2 (11.76%)
Condition/Survival	Alive	4 (23.54%)
	Death (related to the disease)	13 (76.46%)
Reproductive Status	Not Spayed	5 (29.41%)
	Spayed	12 (70.59%)

Table S4. Significant altered pathways for Pathway Analysis by KEGG in the study of feline mammary carcinoma by GC-MS analysis.

Pathway Name	Hits	p-value	FDR	Impact
Alanine, aspartate and glutamate metabolism	5/28	$7.32x10^{-6}$	$5.85x10^{-4}$	0.51
Glyoxylate and dicarboxylate metabolism	4/32	$3.04x10^{-4}$	$9.68x10^{-3}$	0.22
Butanoate metabolism	3/15	$4.70x10^{-4}$	$9.68x10^{-3}$	0.03
Arginine and proline metabolism	4/36	$4.84x10^{-4}$	$9.68x10^{-3}$	0.09
Starch and sucrose metabolism	3/18	$8.26x10^{-4}$	$1.32x10^{-2}$	0.0

Table S5. Significantly altered metabolites by analysis of variance (ANOVA) for comparison between control and different clinical stages of CMF.

Metabolite	F-Value	P-Value	FDR	Tukey's HSD
Ribonic acid	14.29	$8.18x10^{-5}$	0.01	I-II-Control; III-IV-Control
Aspartic acid	11.24	$3.59x10^{-4}$	0.01	III-IV-Control
4-Hidroxyproline	10.14	$6.43x10^{-4}$	0.01	I-II-Control; III-IV-Control
Methionine sulfoxide	9.70	$8.16x10^{-4}$	0.01	I-II-Control; III-IV-Control
Glutamic acid	9.61	$8.61x10^{-4}$	0.01	III-IV-Control
Proline	6.96	$4.13x10^{-3}$	0.04	I-II-Control; III-IV-Control
Galactinol	6.88	$4.35x10^{-3}$	0.04	I-II-Control; III-IV-I-II
Eritronic acid	6.61	$5.15x10^{-3}$	0.04	I-II-Control; III-IV-Control

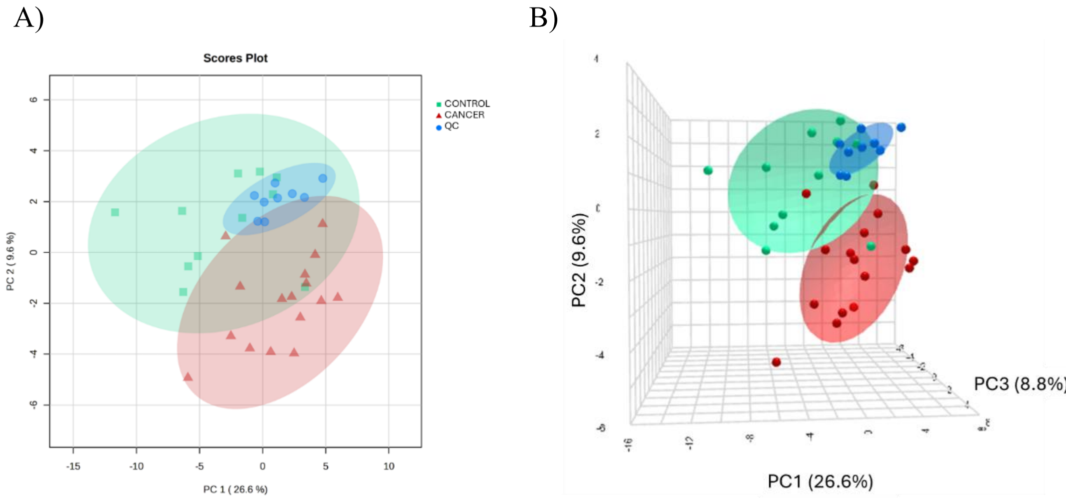

Fig. S1. PCA models built in MetaboAnalyst 6.0. for normalized data **A)** 2D score plot **B)** 3D score plot. Statistical parameters of the model: ANOVA F-value = 9.4693 and p-value = 0.002. Labels: CANCER, red triangles; CONTROL, green squares; and QC (Quality Controls), blue dots.

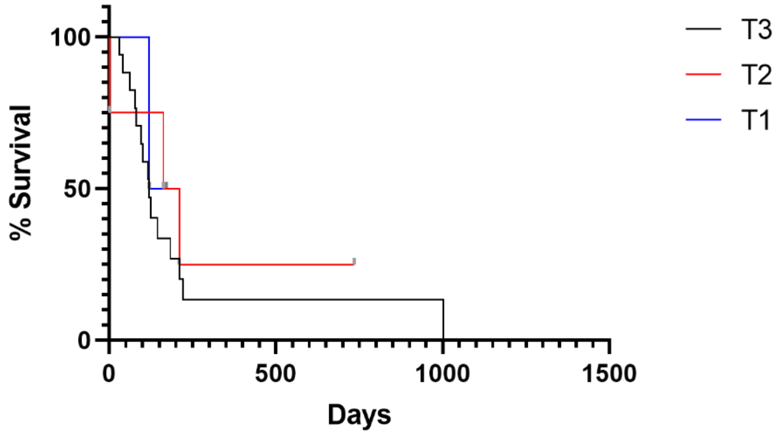

Fig. S2. Kaplan-Meier survival curves of female cats with mammary carcinoma according to tumor size classification. T means tumor size, T1: T < 2 cm; T2: 2 cm > T > 3 cm; and T3: T > 3 cm.
